# DREAMeR: Drug use Reconsidered in the Elderly using goal Attainment scales during Medication Review; study protocol of a randomised controlled trial

**DOI:** 10.1186/s12877-018-0877-1

**Published:** 2018-08-24

**Authors:** Sanne Verdoorn, Henk-Frans Kwint, Jeanet Blom, Jacobijn Gussekloo, Marcel L. Bouvy

**Affiliations:** 1 Division of Pharmacoepidemiology and Clinical Pharmacology, Utrecht Institute for Pharmaceutical Sciences, Utrecht University, Utrecht, The Netherlands; 2 SIR Institute for Pharmacy Practice and Policy, Leiden, The Netherlands; 3 Department of Public Health and Primary Care, Leiden University Medical Centre, Leiden, The Netherlands; 4 Department of Internal Medicine, section Gerontology and Geriatrics, Leiden University Medical Centre, Leiden, The Netherlands

**Keywords:** Clinical medication review, Elderly, Polypharmacy, Goal attainment scaling, Randomised controlled trial, Pharmacists, Primary care

## Abstract

**Background:**

Clinical medication reviews (CMR) are increasingly performed in older patients with polypharmacy. Studies have shown positive effects of CMR on process- and intermediate outcomes, like drug-related problems (DRPs). Little effect has been shown on clinical outcomes, like hospital admissions or health-related quality of life (HR-QoL). In particular, HR-QoL is related to the individual health-related goals and complaints of patients. The aim of this study is to investigate the effects of a CMR focused on personal goals on HR-QoL and health-related complaints in older patients with polypharmacy.

**Methods:**

A randomised controlled trial will be performed in 35 Dutch community pharmacies aiming to include 630 patients aged 70 years and older using seven or more chronic drugs. Patients will be randomly assigned to control or intervention group by block-randomisation per pharmacy. Patients in the intervention group receive a CMR focused on patients’ preferences, personal goals and health-related complaints. With every goal a goal attainment scale (GAS) will be proposed. Primary outcome measures are HR-QoL, measured with the EQ-5D-5L and EQ-VAS and the number of health-related complaints per patient measured with a written questionnaire, during a follow-up period of six months. Secondary outcomes are healthcare utilisation, number and type of drug changes, number and type of health-related goals, scores on GAS and number and type of DRPs and interventions.

**Discussion:**

This study is expected to add evidence on the effects of a CMR on HR-QoL and health-related complaints in older patients with polypharmacy. New in this study is the use of personal goals measured with GAS and health-related complaints as patient-related outcome measures.

**Trial registration:**

Netherlands Trial Register; NTR5713.

## Background

The ageing population and increased availability of evidence on the potentially beneficial effects of preventive medicine has led to a continuous increase in the number of older patients on chronic drug treatment. Especially in the last decades of life drug use is increasing fast [1]. More than one fifth of patients aged 65 years or older is using five or more medicines and almost one out of four older patients with polypharmacy has potential inappropriate medication [2, 3]. The health care costs of inappropriate use of medicines are likely to be high, mainly due to drug-related hospital admissions [4, 5]. Because of the changing health status in older people, the potential consequences of inappropriate medication and new insights in therapy, chronic medication use must be reviewed regularly [6, 7].

Clinical medication reviews (CMRs) are increasingly performed over the last years [8–13]. In the Netherlands pharmacists and GPs are expected to perform regular CMR in older patients according to the Healthcare inspectorate and national guidelines [7]. Many studies have shown effects of CMR on the quality of drug therapy, like reducing Drug-Related Problems (DRPs) and inappropriate prescribing [12, 14–17]. Also studies have shown beneficial effects on disease specific outcomes, like reducing LDL-cholesterol or HbA1c [15, 18, 19]. Moreover, studies suggest that CMR can improve more specific outcomes such as pain management and reduction of falls [9, 20, 21]. However, little effect has been shown on major clinical outcomes, like morbidity, mortality, hospital admissions and health-related quality of life (HR-QoL) [6, 8, 20, 22–25]. Only one study has shown that medication review with follow-up service improves HR-QoL and is likely to be cost-effective [26]. However, the extent of time and the frequency of follow-up contacts in this study is not common for CMR. Therefore extrapolation of the study results is difficult.

According to recent systematic reviews, future studies investigating CMR should be high quality studies including high-risk patients and using relevant outcome measures. CMR should be more targeted on problems that patients experience themselves and outcome measures should be more patient-related [25, 27]. Focusing on patient’s preferences, health-related complaints and personal goals could be a way to improve patients’ HR-QoL. Little is known about the incidence of health-related complaints in older people and to what extent these complaints are related to the HR-QoL of older patients. The only patient-reported complaints that have yet been studied are pain and falls [25, 27, 28]. The effect of CMR on other health-related complaints like dizziness, tiredness or intestinal problems has not been studied.

One way of establishing a patient-centred approach in CMR is by setting personal goals. Attainment of goals can be evaluated by Goal Attainment Scales (GAS). This instrument is used to measure progress on patient specific health-related goals. GAS has been previously used in rehabilitation care and is increasingly used for studies in (frail) older people [29, 30]. However, this tool has never been used in CMR before. Goal setting in CMR can be used a part of a shared-decision making process to reach optimal therapy for patient’s current situation, to prioritize the most important problems for the patient, with the aim to eventually improve patient’s HR-QoL. An example of an expected health-related goal suggested by a patient during a CMR could be the wish to reduce pain. The severity of pain could be easily measured on the Visual Analogue Scale (VAS) and a GAS could be proposed. During the CMR the pain medication could be optimized to achieve this goal. Another expected goal of older people with polypharmacy could be the wish to use less medication. This could be an excellent opportunity for the pharmacist and GP to address “deprescribing” - the act of tapering, reducing or stopping a medication – and thereby balancing the benefits of the drug (e.g. long term effect) against the disadvantages (e.g. experienced adverse effects) [31]. Also in the perspective of reducing health-related complaints, which could be related to possible side effects of medication, “deprescribing” could be addressed. Current studies on this topic indicate that it is possible to discontinue (preventive) medication in older people and that reducing the number of medicines may decrease adverse events and improve quality of life [32, 33]. The drawback of using GAS in a randomised controlled trial is that, as the application of GAS is part of the intervention, attainment of personal goals can only be measured in the intervention group. This study therefore chose HR-QoL as primary outcome as we expect attainment of personal goals will improve quality of life.

In the DREAMeR-study we developed a patient-centred approach of CMR. The aim of this randomised controlled trial is to determine the effect of a CMR focusing on the patient’s preferences, health-related complaints and personal goals related to their medication on patients’ health-related quality of life and their health-related complaints.

## Methods

### Study design and setting

The study is a randomised controlled trial performed in 35 community pharmacies spread throughout the Netherlands. The design, conduct, and reporting of the DREAMeR will adhere to the Consolidation Standards of Reporting Trials (CONSORT) guidelines [34] and basic requirements from the Standard Protocol Items: Recommendations for Interventional Trials (SPIRIT) [35]. The intervention consists of a CMR performed by a community pharmacist in collaboration with a general practitioner (GP). Participants in the control group will be placed on a waiting-list; they receive a CMR after the study period (postponed intervention). Patients will be followed-up for six months.

**Fig. 1:**
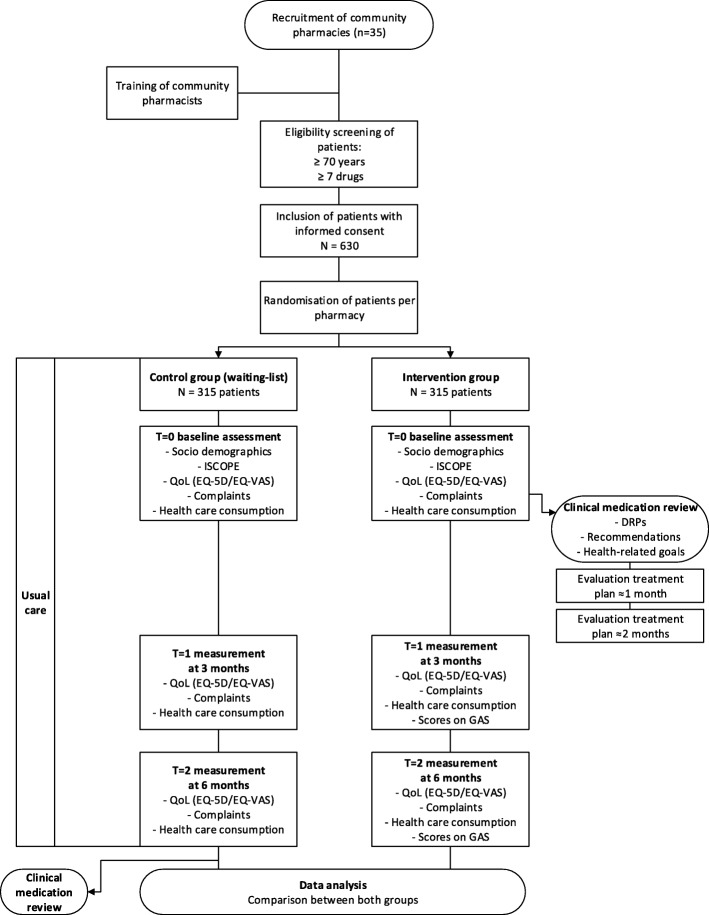
Study design DREAMeR-study

The flowchart of Fig. 1 provides a schematic overview of the study phases along with the participant flow at each study phase.

### Pharmacists

The study was conducted at Dutch community pharmacy franchisees of “Service Apotheek”. Participating community pharmacists must be accredited for CMR before start of the study and have performed at least 25 CMRs annually over the past three years. These accredited CMR courses consist of eight course days, video conferences and the obligation to present a portfolio with a number of medication reviews. Finally, the pharmacists should have agreement with at least one GP to join CMR. One or two community pharmacists will conduct the CMRs in each pharmacy. As one community pharmacy is possibly cooperating with several general practices, the pharmacist will collaborate with different GPs.

All participating pharmacists must attend a training day prior to the study. During this training, the pharmacists will be instructed about every aspect of the study, like registration, data collection, using GAS during CMR and formulating SMART (specific, measurable, acceptable, realistic and time bound) goals together with patients. During the study, monthly web-conferences will be organized, where the pharmacists will present a CMR case with specific attention to the use of GAS.

### Participants

The following inclusion and exclusion criteria are defined:

#### Inclusion criteria

- Community dwelling patients aged 70 years or older.
- Use of seven or more chronic oral drugs. Chronic use of at least one drug is defined as at least three dispensing moments for three months in the last 12 months.

#### Exclusion criteria

- An expected life expectancy shorter than six months.
- A hospital admission within one month before the inclusion date.
- A received CMR in the past 12 months.
- GP is not the primary caregiver (patients receiving repeat prescriptions solely from a specialist).

### Recruitment

The participants will be recruited by their community pharmacists. We expect that in each pharmacy approximately 300–400 patients will be eligible for the study based on the age and number of drugs. We expect each pharmacy to include about 20–30 patients. With an expected response rate of 25%, community pharmacists will invite a total of 50–100 participants per pharmacy.

To avoid selection bias, the inclusion procedure consists of different steps. At first, all patients are screened for the inclusion criteria by the pharmacist. The pharmacist sends the lists with the selected patients to their GPs. The GPs judge the patients on the exclusion criteria and sends the list back to the pharmacist. An anonymous but numbered list is sent by the pharmacist to the researcher to randomly assign 50 patients which will be invited first. Patients are then invited by letter and/or telephone consultation by their pharmacist.

### Randomisation

Randomisation will be performed on patient level per pharmacy. To obtain equal numbers of patients in the intervention and the control group per pharmacy, we use block-randomisation. A block consists of the number of patients who agreed to participate in a pharmacy, usually about 20–30 patients. If an initial inclusion results in less than 20 patients, a second invitation round and block-randomisation will take place. The randomisation procedure will be executed using a computer generated list of random numbers.

### Blinding

Participants, pharmacists and GPs cannot be blinded due to the nature of the intervention. All the results will be collected by the researcher in a database. This database will be handed to a statistician who will conduct a blinded analysis to prevent bias in the evaluation of the outcome measures.

### Sample size calculation

The sample size is based on an expected change on the EQ-5D utility score of 0.05 ± 0.20 over six months. This difference is considered to be a clinically relevant and feasible, based on previous studies in Spain and the Netherlands [15, 26].

To achieve a statistically significant difference in the utility on the EQ-5D with alpha = 0.05 and beta = 0.20, a group size of 252 is sufficient. Allowing for a potential drop-out rate of 25%, a total number of ∼ 630 participants are needed (315 in each group).

This sample size is also expected to be sufficient for the second primary outcome measure: the number of health-related complaints per patient. Because comparative studies with this outcome measure are lacking, we have made some assumptions. If the study population consists of 252 patients per group, a difference on the number of health-related complaints with approximately 0.5 ± 2 with alpha = 0.05 and beta = 0.20 could be demonstrated. We expect that a patient has an average of two health-related complaints with moderate to severe impact on daily life, which may possibly be reduced by 25%. We consider this difference as feasible and clinically relevant. The number of complaints will be highly variable. Therefore we assume a standard deviation of two.

### Intervention

The CMR will be a comprehensive evaluation of patient’s medicines, performed according to an implicit method described in the multidisciplinary guideline “Polypharmacy in the elderly” [7]. This implicit method is called the STRIP-method (Systematic Tool to Reduce Inappropriate Prescribing) and consists of different five steps: i) the CMR starts with a patient interview by the pharmacist. Prior to this interview, the patient completes the ISCOPE (Integrated Systematic Care for Older People) questionnaire, the EQ-5D questionnaire and the questionnaire ‘common complaints in older people’ (see outcome measures for explanation). These questionnaires can be used by the pharmacist during the interview. The pharmacist explores the perceived health complaints that may be related to the medication. All medications in use (including Over The Counter (OTC) medication) by the patient will be discussed. Specific questions will be asked about the experiences of the patient with each drug. Explicit attention will be paid to the practical problems of drug use, effectiveness, adherence and possible side effects. The pharmacist and patient attempt to formulate personal health-related goals (GAS). These goals will concentrate on improving activities of daily living and health-related complaints. ii) After that, DRPs will be identified using all clinical data (laboratory values and diagnoses), medication data (drug dispensing records from the pharmacy) and patient data from the interview. Recommendations will be formulated to solve these DRPs. Complete discontinuation or dose reduction of medication (“deprescribing”) will be addressed when possible. iii) The pharmacist will discuss the DRPs and health-related goals with the GP. A pharmaceutical care plan will be formulated which include which actions will be carried out when and by whom. In this care plan all potential DRPs will be included, focused on patient’s preferences and goals, but also on inappropriate prescribing and prescribing omissions. iv) This pharmaceutical care plan will be then discussed with the patient by the pharmacist or the GP and the actions will be implemented gradually. v) Finally two follow-up moments will be scheduled (within approximately three months). The pharmacist and GP agree on how to perform the discussion of the pharmaceutical care plan with the patient and the follow-up and monitoring. Also the pharmacy technician or practice nurse could be involved in this process. If necessary, the pharmaceutical care plan will be adjusted in concordance with patient, pharmacist and GP. The implementation of the pharmaceutical care plan including follow-up is expected to be completed within approximately three months, depending on the type of interventions and DRPs.

### Data collection

Measurements by means of paper questionnaires and telephone interviews will be carried out at baseline, and at three and six months. It is expected that some patients will experience difficulties completing the questionnaires. These patients may be supported by their family or their pharmacist could ask a research assistant to telephonically guide them through the questionnaire. Characteristics of medication and changes in medication will be assessed using drug dispensing data from the pharmacist. All process outcomes of the CMRs, will be collected using the Service Apotheek Medication Review Tool (SAMRT), a software program designed to record DRPs and interventions [12]. All demographic characteristics (sex, age, ethnicity, marital status), and number of medications at baseline will be recorded at baseline. In addition the ISCOPE (Integrated Systematic Care for Older People) - questionnaire will be completed at baseline [36, 37]. This screening questionnaire contains questions on four domains of health: a functional, somatic (health and illness), mental and social domain. Individuals with problems on three as well as four domains are classified as having complex problems [37].

### Outcome measures

#### Primary outcome measures

The primary outcome measures are the health-related quality of life (HR-QoL) and the number of health-related complaints per patient. These outcome measures are collected at baseline, and at three and six months. HR-QoL will be determined by the EQ-5D-5L and EQ-VAS. The EQ-5D-5L has been shown to be valid and reliable in a variety of populations and patient groups [38]. Utilities will be calculated with the aid of EQ-5D tariff [39]. Quality-adjusted life years (QALYs) will be calculated using linear interpolation between time points. Higher QALY scores indicate more improvement in HR-QoL. An additional cognition-question (EQ-6D) will be included in the questionnaire, but will not be used to calculate utilities and QALYs, as no tariff exists for the EQ-6D. [38]

**Fig. 2:**
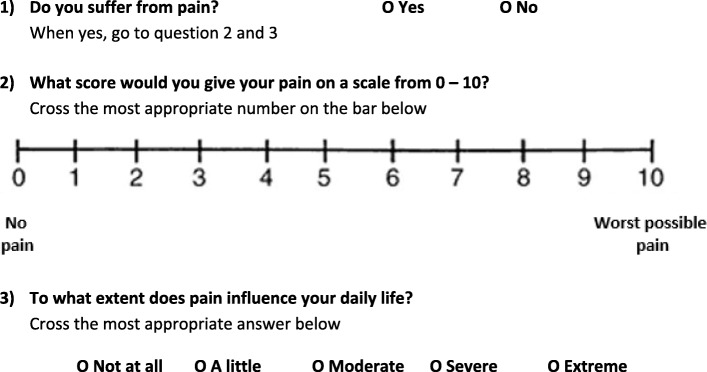
Example of question about the complaint “pain”

The number and severity of health-related complaints will be assessed using a self-developed questionnaire which is based on common adverse effects of drugs and common complaints in older people previously identified in the ISCOPE-study (see Table 1 for all the complaints that will be measured) [40]. A complaint will be scored on severity with the VAS and on influence on daily life with a five-point Likert scale to determine the number of complaints with moderate to severe impact on patient’s daily life (see example in Fig. 2).

**Table 1:** Health-related complaints measured in the questionnaire

Type of health-related complaint	
Pain	
Itching	
Dyspnoea	
Problems with walking (mobility)	
Dizziness	
Drowsiness/sedation	
Intestinal complaints (constipation/diarrhoea)	
Gastric complaints (reflux or ulcer)	
Forgetfulness	
Fatigue	
Dry mouth	
Incontinence	
Other

#### Secondary outcome measures

Secondary outcome measures are health care consumption and number and type of drug changes. Health care consumption will be measured at baseline and at three and six months with the Dutch Medical Consumption (*i*MTA) Questionnaire including informal care [41]. Healthcare utilisation will be valued according to guidelines for economic evaluation in healthcare in the Netherlands [42]. Data about number and type of drugs in use will be derived from the drug dispensing records from the pharmacy information system over a period from 24 months before the start of the study until nine months after the start of the study. Use of OTC drugs is not always recorded in drug dispensing data because the majority is purchased outside the pharmacy in so called drugstores.

Other secondary outcomes are process outcomes of the CMRs. The prevalence of number and type DRPs will be measured at baseline. DRPs will be classified accorded to an adapted version of Hepler and Strand which is described in the STRIP-method [7, 43]. Also the interventions, like drug changes, will be validated with the drug dispensing data. Different DRP-types and interventions are summarized in Table 2. Next to the interventions, the implementation rates will be calculated, defined as the percentage of the recommendations that are fully or partly accepted by GP and patient (e.g. dose change when cessation of drug was proposed).

**Table 2:** Overview of different DRP and intervention types

DRP type	Intervention type
Overtreatment	Drug added
Drug not effective	Drug ceased
Suboptimal therapy	Drug replaced
(potential) Adverse effect	Dosage regimen changed
Dose too high	Dosage form changed
Dose too low	Performed monitoring
Usage problem	Information/advice provided
Clinical relevant contra-indication	Medication synchronized
Clinical relevant interaction	Other
	No Intervention

Health-related goals with GAS assessed by the pharmacist and the patient during the patient interview will be recorded in a separate database. At the start of the study a database with 50 health-related goals with GAS was composed to help the pharmacists with common examples. This list will be further expanded during the study based on the health-related goals people propose. GAS should be formulated SMART: specific, measurable, acceptable, realistic and time-bound. The number and type of health-related goals will be assessed at baseline. The scores on the GAS (− 3 to + 2) will be assessed by telephonic interviews at three and six months. An example of a GAS can be found in Table 3.

**Table 3:** Example goal attainment scale

Problem	Goal		Plan	Evaluation
Pain	Reduce pain from VAS-score 6 to VAS-score 4		Start with painkillers; e.g. paracetamol in accurate dose	After 2-4 weeks
Was the goal achieved?		Description	Example	Score
	Yes?	A lot more	No pain anymore or VAS-score < 3	+2
		A little more	Pain VAS-score 3	+1
		As expected	Pain VAS-score 4	0
	No?	Partially achieved	Pain VAS-score 5	-1
		No change	Pain VAS-score 6	-2
		Got worse	Pain VAS-score >6	-3

All outcomes will be assessed at patient level. An overview of all the outcome measures and instruments is shown in Table 4.

**Table 4:** Overview outcome measures DREAMeR study

Parameters	Instrument or data source
Baseline assessment	
Socio-Demographics	Data questionnaire
Complex-problems	ISCOPE questionnaire
Number and type of medication	Extraction dispensing records pharmacy
Type of personal goals (intervention group)	Assessed by pharmacist and patient
Primary outcomes	
Health-related quality of life	EQ-5D-5L and EQ-VAS
Health-related complaints	Self-developed data questionnaire
Secondary outcomes	
Health care consumption	Dutch Medical Consumption (*i*MTA) Questionnaire
Number of changed drugs (drugs added and ceased)	Extraction dispensing records pharmacy
Scores on Goal attainment Scales (intervention group)	
Drug-Related Problems (intervention group)	Recorded in the SAMRT with encodings of Table 2
Proposals and interventions of the pharmaceutical care plan (intervention group)	Recorded in the SAMRT with encodings of Table 2

### Statistical analyses

Descriptive statistics will be used for patient characteristics. Dropout and loss to follow-up will be described. Effect analyses will be performed according to both ‘intention to treat’ and ‘per protocol’ principles. Longitudinal differences in the primary outcomes between the two groups will be analysed with linear and logistic mixed model analyses. Intervention, time (baseline, and at three and six months), and the interaction between intervention and time will be used as fixed factors in the linear mixed model. Participant identification number will be included as a random effect to account for the dependence of repeated observations. Baseline characteristics can be integrated into the mixed model to control for confounding. Secondary outcomes are analysed analogously. Explorative subgroup analyses will be performed. In case of missing data, sensitivity analyses will be conducted to examine the influence of missing data on the study findings. *p*-Values ≤0.05 will be considered significant.

### Economic evaluation

An economic evaluation will be added to investigate the additional costs per QALY. Costs will be measured from a health care perspective. Lost productivity costs will not be included since we expect that all patients will be over 65 years of age and therefore retired. Healthcare costs will be assessed using the Dutch Medical Consumption (*i*MTA) Questionnaire [41]. The costs of the intervention will be calculated by multiplying the time spent by the pharmacist with the average wage of a pharmacist. The time spent by the pharmacist is calculated by the average time writing per CMR for every pharmacy. All pharmacists will be asked to record the time spent for every step of the medication review process; including patient interview, DRP analysis, conversation with GP and follow-up and monitoring. Also the time spent by the pharmacy technician and GP will be recorded. Drug spending will be derived from the pharmacy information system. Calculation of OTC drugs costs will not be possible, because purchase of OTC drugs is often not recorded in the pharmacy information systems. Quality Adjusted Life Years (QALY’s) are used as the measure of effect. They are calculated using the Dutch tariff and the EQ-5D results from the trial. The incremental costs per QALY will be determined. Deterministic and probabilistic sensitivity analyses will be performed.

### Process evaluation

Additionally quantitative (process documentation instruments) and qualitative (semi-structured interviews with pharmacists, patients and GPs) process evaluation will be conducted to identify possible barriers of implementation. The process evaluation involves assessing the extent to which the intervention is performed according to the protocol of the study and the opinion of the participants on the intervention.

### Ethics and confidentiality

The study design, study protocol, procedure and informed consent are approved by the Medical Ethics Committee of the University Medical Centre of Utrecht (protocol number 15/737). Participation is voluntary and all participants will sign informed consent.

### Trial status

Patient recruitment and baseline collection were conducted between April 2016 – February 2017. Outcome data were collected between June 2016 and August 2017. At the time of the initial submission of the manuscript the data collection was still ongoing. First results are expected at the end of 2018.

## Discussion

The DREAMeR-study aims to determine the effects of a CMR on patients’ health-related quality of life and health-related complaints in older people with polypharmacy. What this study adds is the introduction of personal goals measured with goal attainment scales and number of health-related complaints as more patient-related outcome measures. The intervention is a CMR with a patient-centred approach, focusing on patient’s preferences, health-related complaints and personal goals. The patients with a CMR are compared to patients in the control group who receive usual care and will receive a CMR after completion of the study.

The DREAMeR-study was designed to elucidate the potential effects of CMR on clinical outcomes. Several reasons may contribute to the fact that clinical outcomes of CMR are still sparse despite a body of evidence on the effects on a reduction of DRPs. First, given the small baseline prevalence of hospitalisations and mortality very large sample sizes are needed to determine an effect of CMR on these outcomes. This would need budgets that are generally not available for pragmatic practice based studies. A second reason could be that selection criteria for eligible patients were not specific enough. Selection criteria for patients receiving CMR in studies were often set on patients aged 65 years or older using five or more drugs. Probably a large proportion of this sample of patients will have a high baseline HR-QoL and less medication to change, which makes it difficult to show an effect on HR-QoL. A third reason could be that the performed interventions during CMR are very heterogeneous, from adding statins (preventive therapy) to adding painkillers (reducing complaints) and from monitoring renal functions (prevent harm from wrong dosage of ACE-inhibitors) to changing dosage regimens (to improve patient adherence). This could make it difficult to measure an effect on a generic outcome such as HR-QoL. Finally, it is possible that in previous studies not all involved health care providers had sufficient experience with CMR, which makes it difficult to perform a good CMR with an effect for the patient. Specific guidelines for CMR have been developed, but compliance with these guidelines in daily practice is likely to be suboptimal.

Taken all these above mentioned hypotheses into account, we have designed the protocol for the DREAMeR study. Despite the fact that it is difficult to prove an effect of CMR on HR-QoL, we still chose the EQ-5D to be one of the primary outcome measures in this study. In our opinion, this is one of the most important outcome measures for older patients. Another advantage of measuring the EQ-5D is that it gives the opportunity to perform a cost-effectiveness analysis, which is needed for health care policies. We think that addressing the complaints and goals of patients in the DREAMeR-study may translate in increased HR-QoL.

Other studies suggest the importance of more patient-related outcomes in CMR [25, 27]. Therefore in this study a second primary outcome measure will be investigated next to the HR-QoL, defined as the number of health-related complaints with impact on patient’s daily life. Previous studies also suggest that CMR might be more beneficial for more specific patient groups. We aim to improve the patient selection with stricter selection criteria: patients aged 70 years or older and using seven or more chronic drugs. With these criteria, more frail patients with more complex diseases will be selected. These patients are potentially more likely to benefit from a medication review and more attention could be paid to the dilemma of “deprescribing” for patients who experience more negative than positive effects of drugs or patients who wish to use less medicines [44]. Until now it is not possible to select frail persons directly from pharmacy information systems. That is why we choose to increase age and number of drugs.

The wish for the reduction of severe complaints or the number of medicines, can be translated into goals. By proposing personal goals with the patient, the interventions in the pharmaceutical care plan can be prioritized. The most important issues for the patient will get the most attention. Personal goals can be measured with GAS. Older community dwelling persons with complex problems are able to set personal goals using GAS according to one study [29]. GAS is a patient-centred outcome measure that cannot only demonstrate a change in health and function, but can also be scaled to allow for comparison of change within and between groups of older adults with distinct personalized goals [45–47]. The use of GAS makes it possible to aggregate the heterogeneous interventions during CMR. This is a different approach compared to the usual process-outcomes that are measured in CMR. However, GAS is a new concept for both community pharmacists and GPs to work with. To support pharmacists with the application of GAS during CMR, we offer a training day, monthly webconferences and a helpdesk service.

After the performance of the patient interview and the preparation of the pharmaceutical care plan in consultation with the GP and patient, specific attention will be paid to the follow-up of the interventions and monitoring of patients during the CMR. This may lead to a higher implementation rate of the interventions. [48].

Besides the use of more patient-related outcome measures and a patient-centred approach of CMR with specific attention to follow-up, the training, support and selection of the pharmacists is another strength of this study. We have selected pharmacists with previous experience and training in CMR and good collaboration with their GPs. We consider these conditions essential to perform a good CMR. However, we still provided extra training and monthly webconferences to ensure a good implementation of the study protocol and gain experience in working with GAS. Finally, a process-evaluation will give insight in the facilitators and barriers for implementation.

Although many have studies evaluated the effect of CMR on DRPs, we are of the opinion that the innovative approach chosen for CMR in this study will give us more insight into ‘what really matters to the patient’.

## Abbreviations

CMR: Clinical Medication Review
CONSORT: Consolidated standards of reporting trials
DRP: Drug Related Problems
GAS: Goal Attainment Scale
GP: General Practitioner
HR-QoL: Health-Related Quality of Life
ICER: Incremental Cost-Effectiveness Ratio
QALY: Quality-Adjusted Life Year
SAMRT: Service Apotheek Medication Review Tool
STRIP: Systematic Tool to Reduce Inappropriate Prescribing
VAS: Visual Analogue Scale
